# Increased chest CT derived bone and muscle measures capture markers of improved morbidity and mortality in COPD

**DOI:** 10.1186/s12931-022-02237-w

**Published:** 2022-11-15

**Authors:** Ava C. Wilson, Jessica M. Bon, Stephanie Mason, Alejandro A. Diaz, Sharon M. Lutz, Raul San Jose Estepar, Gregory L. Kinney, John E. Hokanson, Stephen I. Rennard, Richard Casaburi, Surya P. Bhatt, Marguerite R. Irvin, Craig P. Hersh, Mark T. Dransfield, George R. Washko, Elizabeth A. Regan, Merry-Lynn McDonald

**Affiliations:** 1 Department of Epidemiology, School of Public Health, University of Alabama at Birmingham, 701, 19th Street S., LHRB 440, Birmingham, AL 35233 USA; 2 Division of Pulmonary, Allergy and Critical Care Medicine, Department of Medicine, University of Alabama at Birmingham, Birmingham, AL USA; 3 Division of Pulmonary, Allergy and Critical Medicine, University of Pittsburgh Health System, Pittsburgh, PA USA; 4 VA Pittsburgh Health System, Pittsburgh, PA USA; 5 Division of Pulmonary and Critical Care Medicine, Brigham and Women’s Hospital, Boston, MA USA; 6 Department of Radiology, Brigham and Women’s Hospital, Harvard Medical School, Boston, MA USA; 7 Department of Epidemiology, University of Colorado Anschutz Medical Campus, Aurora, CO USA; 8 Department of Medicine, Nebraska Medical Center, Omaha, NE USA; 9 Rehabilitation Clinical Trials Center, Lundquist Institute for Biomedical Innovation at Harbor Harbor-UCLA Medical Center, Torrance, CA USA; 10 Channing Division of Network Medicine, Brigham and Women’s Hospital, Boston, MA USA; 11 Department of Medicine, National Jewish Hospital, Denver, CO USA; 12 Department of Genetics, University of Alabama at Birmingham, Birmingham, AL USA

**Keywords:** COPD, Sarcopenia, Osteoporosis, Screening

## Abstract

**Background:**

Chronic obstructive pulmonary disease (COPD) is a disease of accelerated aging and is associated with comorbid conditions including osteoporosis and sarcopenia. These extrapulmonary conditions are highly prevalent yet frequently underdiagnosed and overlooked by pulmonologists in COPD treatment and management. There is evidence supporting a role for bone-muscle crosstalk which may compound osteoporosis and sarcopenia risk in COPD. Chest CT is commonly utilized in COPD management, and we evaluated its utility to identify low bone mineral density (BMD) and reduced pectoralis muscle area (PMA) as surrogates for osteoporosis and sarcopenia. We then tested whether BMD and PMA were associated with morbidity and mortality in COPD.

**Methods:**

BMD and PMA were analyzed from chest CT scans of 8468 COPDGene participants with COPD and controls (smoking and non-smoking). Multivariable regression models tested the relationship of BMD and PMA with measures of function (6-min walk distance (6MWD), handgrip strength) and disease severity (percent emphysema and lung function). Multivariable Cox proportional hazards models were used to evaluate the relationship between sex-specific quartiles of BMD and/or PMA derived from non-smoking controls with all-cause mortality.

**Results:**

COPD subjects had significantly lower BMD and PMA compared with controls. Higher BMD and PMA were associated with increased physical function and less disease severity. Participants with the highest BMD and PMA quartiles had a significantly reduced mortality risk (36% and 46%) compared to the lowest quartiles.

**Conclusions:**

These findings highlight the potential for CT-derived BMD and PMA to characterize osteoporosis and sarcopenia using equipment available in the pulmonary setting.

**Supplementary Information:**

The online version contains supplementary material available at 10.1186/s12931-022-02237-w.

## Introduction

Chronic obstructive pulmonary disease (COPD) is the fourth leading cause of death worldwide and is typically classified as a disease of accelerated aging [1, 2]. In addition to impaired lung function, patients with COPD experience significantly more extrapulmonary manifestations or comorbidities compared to older adults [3, 4]. These extrapulmonary manifestations include sarcopenia and osteoporosis, which further reduce quality of life and complicate disease management [5, 6]. Although osteoporosis and sarcopenia are common among patients with COPD, they are not routinely diagnosed or monitored by pulmonologists [7, 8].

Sarcopenia is characterized by reduced muscle strength and mass leading to a decline in physical performance [9]. Osteoporosis is characterized by reduced bone strength due to loss of bone mineral density (BMD) and impaired bone architecture that leads to fractures [7]. Further, secretion of myokines and osteokines by bone and muscle enable cross-talk between the tissues influencing bone metabolism and skeletal muscle growth and function [10]. In COPD, reduced muscle mass is associated with worse pulmonary function and increased risk of osteoporosis [11]. Similarly, COPD patients with osteoporosis have worse disease severity than COPD patients without osteoporosis [12]. Treatments for sarcopenia include resistance training, nutrition management, and pharmaceutical therapies and for osteoporosis, treatments include calcium and vitamin D supplementation, weight bearing exercise, fall prevention, and antiresorptive and anabolic medications [13, 14]. Thus, in COPD, both sarcopenia and osteoporosis are potentially treatable [7, 9, 15–19].

Assessment of muscle mass and BMD are not typically evaluated as a part of routine care for COPD. Dual-energy x-ray absorptiometry (DEXA) is considered the gold standard for diagnosis of osteoporosis and is recommended for screening by the United States Preventative Services Task Force (USPSTF) [20, 21]. DEXA can also be used to measure lean mass, bone mass, and fat mass in research settings, but is not routinely used in clinical settings for evaluating muscle mass [17, 22]. Chest computed tomography (CT) scans are used for lung cancer screening in heavy smokers and to assess COPD [17, 22–24]. Quantitative CT can be used to characterize reduced BMD and bone architecture [23, 25, 26]. In addition, DEXA measures of whole body lean mass correlates with PMA derived from chest CT has and have been validated as a useful tool, which can be used to derive measurements of fat free muscle mass index (FFMI) to diagnose and monitor sarcopenia in COPD [22, 24, 27, 28].

Previous studies have shown that CT-based measures of BMD and PMA are associated with disease severity in COPD separately [16, 17, 23, 24, 29]. However, the correlation between BMD and PMA and their contribution to mortality have not been fully explored. To fill this gap, we assessed whether lower PMA and BMD, individually and in combination, in COPD are associated with a higher burden of COPD in terms of clinical and survival outcomes in 4,248 COPD subjects with mild to very severe COPD.

## Methods

### Study population

COPDGene (NCT00608764, www.copdgene.org) is an ongoing multicenter, observational study of the progression and genetic susceptibility to COPD, the details of which have been described previously [30]. COPDGene enrolled Non-Hispanic White and African American current or former smokers with at least a 10 pack-year smoking history, 45 to 80 years of age, who were followed longitudinally. Mild to very severe COPD cases included in this analysis had airflow limitation with post-bronchodilator spirometry GOLD (Global Initiative for Chronic Obstructive Lung Disease) grade 1 or higher at baseline (forced expiratory volume in 1 s [FEV_1_] ≥ 80% predicted and FEV_1_ expressed as a percentage of forced vital capacity [FEV_1_/FVC] < 0.7) [31]. A total of 10,371 individuals were enrolled in the COPDGene study. Of these, 9,703 had both PMA and BMD measurement data obtained from chest CT scans at baseline. Of the participants with baseline PMA and BMD data, 4,248 had mild to very severe COPD (GOLD 1–4), 4,116 smoking controls had normal spirometry (GOLD = 0), and 104 participants were age-matched, non-smoking controls. Participants with COPD were included in the analysis of the association of BMD and PMA with functional and clinical outcomes. Non-smoking controls were utilized as a healthy control group in which we derived sex-stratified quantiles of BMD and PMA used for survival analyses of COPD participants. Of the participants with COPD, 3703 had survival data and were included in survival analyses. In addition, the potential effects of age, smoking, and COPD disease severity on BMD and PMA were assessed by comparing participants with mild to very severe COPD to non-smoking controls and participants with normal spirometry.

Institutional Review Board approval was obtained for this analysis. All COPDGene participants provided written, informed consent, to participate in the study and IRB approval was obtained at each of the clinical centers.

### Measurements

As previously described [24], PMA values were measured in cm^2^ and derived from chest CTs collected at baseline. As described in detail [17], BMD, measured in mg/cm^3^, was estimated from measured bone attenuation values on chest CT scans sampled in the cancellous bone at T6–L1 vertebral levels at baseline. A mean value was calculated and vertebral levels that were fractured were excluded. Using the mean bone attenuation value and prediction equations derived from calibrated volumetric BMD measurements, BMD was calculated for each participant [17]. Vertebrae that were fractured or were otherwise abnormal were excluded from measurements of BMD and attenuation values (N = 6 participants).

Cigarette smoking was quantified in pack years, with one pack year equivalent to smoking 20 cigarettes per day for one year. The number of comorbidities were reported as 3 categories (0 comorbidities, 1 or 2 comorbidities, 3 or more comorbidities) based on the presence of the following as documented by self-report: cancer, diabetes, heart disease, and gastroesophageal reflux disease. BMI was measured in kg/m^2^ and categorized using the WHO cut points of underweight, healthy weight, overweight, and obese (BMI < 18.5, 18.5 ≤ BMI < 25, 25 ≤ BMI < 30, and BMI ≥ 30, respectively). Handgrip strength was measured at visit 3 (10 years from baseline) and is reported as the mean of three measurements made from the participant’s dominant, unsupported hand [32]. Severe exacerbations were defined as a reported emergency room visit and/or hospitalization for an acute episode of respiratory disease.

### Measures of function and disease severity

Measurements for functional outcomes of interest and disease severity included FEV_1_ percent predicted, percent emphysema, handgrip strength, and six-minute walk distance (6MWD) measured in meters. The 6MWD test has been validated as a tool for evaluating the functional status of COPD patients [33]. Percent emphysema was determined based on CT imaging and defined as the total percentage of both lungs with low attenuation values <  − 950 Hounsfield units on inspiratory images [34]. C-reactive protein (CRP) levels were obtained from peripheral venous blood and normalized using a log transformation [35].

### Statistical analysis

Descriptive statistics are presented for continuous variables as mean ± SD and for categorical variables as frequency and percent in participants with COPD and controls (normal spirometry and non-smokers). Multivariable linear regression was used to examine the relationship between CT-derived PMA with BMD in participants with COPD. The multivariable model of PMA as a predictor of BMD was adjusted for pack years, gender, FEV_1_ percent predicted, BMI category, severe exacerbations, and number of comorbidities category. Multivariable linear regression was used to examine the relationship between CT-derived BMD and, separately, PMA with measures of disease severity (FEV_1_ percent predicted and percent emphysema) and function (handgrip strength and 6MWD) in COPD participants adjusting for pack years, gender, number of comorbidities category, severe exacerbations, and BMI category. 6MWD was additionally adjusted for height. Beta coefficients and 95% confidence intervals were computed for adjusted multivariable models. Correlation (r) values were obtained by taking the square root of the adjusted r^2^ obtained from multivariable linear regression models. The relationship between mean BMD and PMA across levels of COPD severity (Global Initiative for Obstructive Lung Disease (GOLD) score) was assessed by one-way ANOVA. Survival modeling was performed using Cox proportional hazards regression to obtain hazard ratios (HR) and 95% confidence intervals (CI) for the risk of all-cause mortality with PMA and/or BMD in participants with COPD. Cox proportional hazards models included adjustment for pack years, gender, FEV_1_ percent predicted, categorical BMI, severe exacerbations, and number of comorbidities. Correlation (r) values from survival analyses were obtained by taking the square root of the adjusted Cox and Snell pseudo r^2^ value obtained from Cox proportional hazards models [36]. All p-values are two-sided with p < 0.05 considered statistically significant. All statistical analyses were performed using R version 3.6.0.

## Results

### Characteristics of study population

Clinical and demographic characteristics of COPDGene study participants included in the present analyses are summarized in Table 1. On average, participants with mild to very severe COPD were older (63.1 ± 8.6 years) and more likely to be male (56.3%) when compared to those with normal spirometry (56.7 ± 8.4 years, 53.3% male,) and non-smoking controls (62.2 ± 9.4 years, 30.8% male) (Table 1). COPD participants and non-smoking controls tended to be overweight (33.8% and 36.5%, respectively), while smoking controls tended to be obese (37.2%) (Table 1). A majority of COPD participants and non-smoking controls had one or two comorbidities (54.8% and 49.0%, respectively), while smoking controls had fewer reported comorbidities (47.8%) (Table 1). On average, COPD participants had the highest levels of emphysema (11.7 ± 12.3%) and lowest lung function (FEV_1_ percent predicted = 57.6 ± 22.7) (Table 1). Participants with normal spirometry had an average percent emphysema of 2.0 ± 2.5% and an average FEV_1_ percent predicted of 97.4 ± 11.4, and non-smoking controls had an average percent emphysema of 1.7 ± 2.2% and an FEV_1_ percent predicted of 102.7 ± 13.6% (Table 1). Participants with COPD had the lowest 6MWD (377.2 ± 124.5 m) compared to participants with normal spirometry (455.7 ± 106.3 m) and non-smoking controls (513.0 ± 100.0 m) (Table 1). On average, handgrip strength was similar across all three study groups (COPD = 28.4 ± 10.6 kg, normal spirometry = 27.8 ± 10.0 kg, non-smoking controls = 27.3 ± 7.1 kg) (Table 1).

**Table 1:** Characteristics of COPDGene study participants

Demographic (mean ± SD)	Mild to very Severe COPD (GOLD 1–4) (N = 4248)	Smoking controls No COPD (GOLD 0) (N = 4116)	Non-smoking controls (N = 104)
Age (years)	63.1 ± 8.6	56.7 ± 8.4	62.2 ± 9.4
Percent Male N (%)	2,393 (56.3)	2,194 (53.3)	32 (30.8)
Race N (%)			
Non-Hispanic White	3,296 (77.6)	2,422 (58.8)	97 (93.3)
African American	952 (22.4)	1,694 (41.2)	7 (6.7)
Pack Years	51.4 ± 27.0	37.3 ± 20.2	–
Current Smoker N (%)	1,833 (43.1)	2,441 (59.3)	–
BMI (kg/m^2^) N (%)			
Underweight (< 18.5)	106 (2.5)	27 (0.66)	1 (0.96)
Healthy Weight (18.5 ≤ BMI < 25)	1,369 (32.2)	1,029 (25.0)	31 (29.8)
Overweight (25 ≤ BMI < 30)	1,435 (33.8)	1,530 (37.2)	38 (36.5)
Obese (BMI ≥ 30)	1,338 (31.5)	1,530 (37.2)	34 (32.7)
Inhaled Corticosteroid Use N (%)	1,880 (45.0)	224 (5.5)	0 (0)
Severe Exacerbations N (%)	818 (19.3)	174 (4.2)	0 (0)
Number of Comorbidities N (%)			
0	1,374 (32.4)	1,965 (47.8)	49 (47.1)
1–2	2,325 (54.8)	1,883 (45.8)	51 (49.0)
3 +	545 (12.8)	266 (6.5)	4 (3.8)
Percent Emphysema	11.7 ± 12.3	2.0 ± 2.5	1.7 ± 2.2
FEV1 Percent Predicted	57.6 ± 22.7	97.4 ± 11.4	102.7 ± 13.6
Six Minute Walking Distance (m)	377.2 ± 124.5	455.7 ± 106.3	513.0 ± 100.0

BMI, body mass index; COPD, chronic obstructive pulmonary disease; FEV1, forced expiratory volume in 1 s; SD, standard deviation

### Measures of BMD across study groups and COPD disease severity

**Fig. 1:**
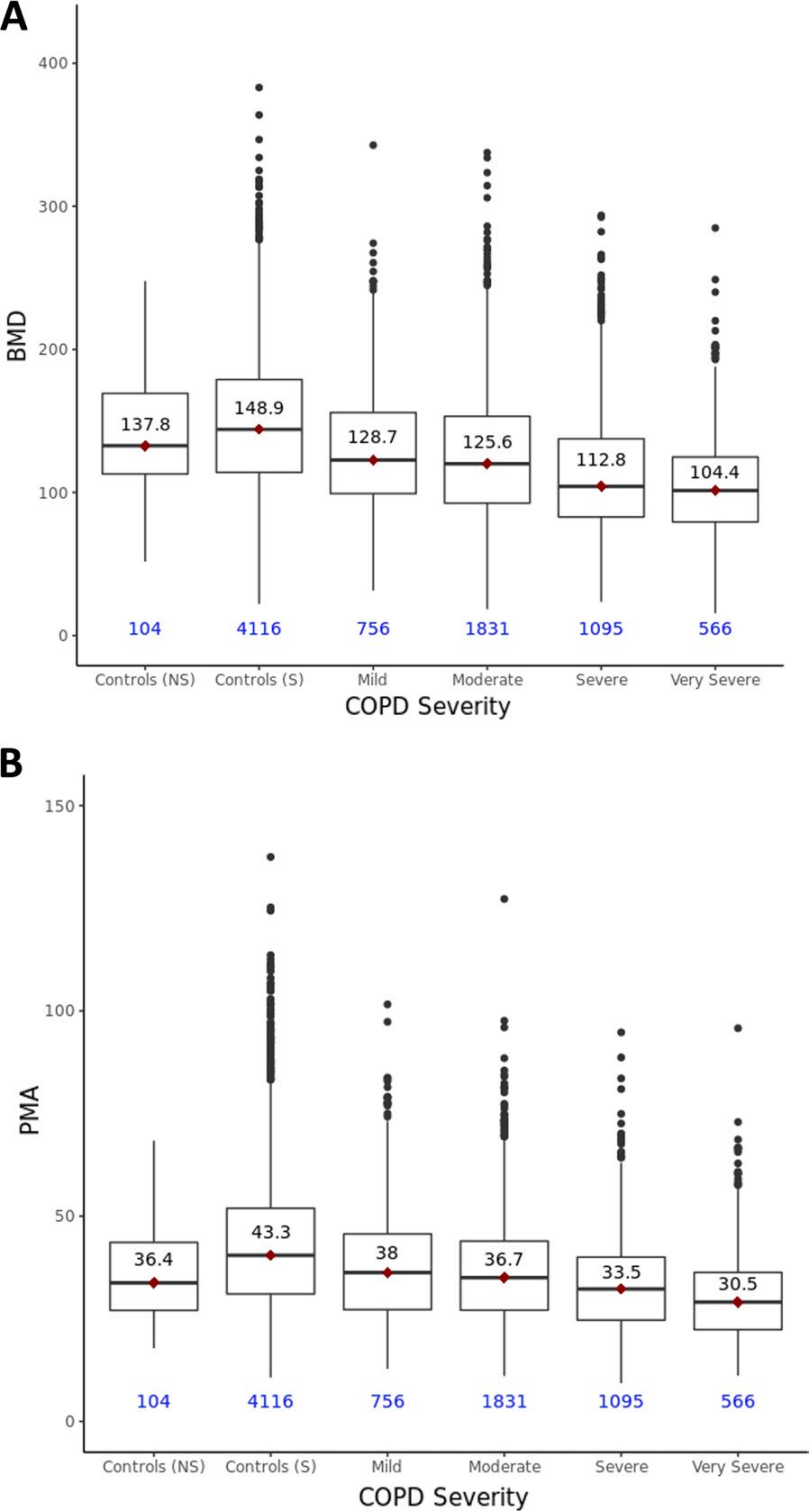
Distribution of BMD (**A**) and PMA (**B**) values with respect to control and COPD status. Control groups included both never smoker (NS) and current/former smoker (S) controls. COPD participants are further categorized by severity using GOLD Stage. Number of subjects in each group is presented below in blue. Median BMD and PMA values are listed in black

There was a significant difference between BMD across all levels of COPD disease severity (p < 0.001). Participants with worse COPD severity tended to have lower BMD (Fig. 1). Specifically, in COPD subjects with mild to very severe COPD (GOLD 1–4), the mean BMD was 120.1 ± 44.4 g/cm^3^. Whereas smoking controls had a mean BMD of 148.9 ± 47.8 g/cm^3^ and non-smoking controls had a mean BMD of 137.8 ± 39.6 g/cm^3^ (Fig. 1). In regression models, having very severe COPD was significantly associated with lower BMD when compared to both non-smoking controls (β = − 33.4, SE = 4.9, p < 0.001) and smoking controls (β = − 44.5, SE = 2.0 p < 0.001). Sex-stratified regression models exhibited a similar trend. In males, having very severe COPD was significantly associated with lower BMD when compared to both non-smoking controls (β = − 43.6, SE = 8.3, p < 0.001) and smoking controls (β = − 48.7, SE = 2.6, p < 0.001). Likewise, in females, having very severe COPD was significantly associated with lower BMD when compared to both non-smoking controls (β = − 26.6, SE = 6.3, p < 0.001) and smoking controls (β = − 39.0, SE = 3.3, p < 0.001).

### Measures of PMA across study groups and COPD disease severity

Similarly, to BMD, there was a significant difference between PMA across all levels of COPD disease severity (p < 0.001). Participants with worse COPD severity tended to have lower PMA (Fig. 1). Specifically, in mild to very severe COPD subjects (GOLD 1–4), the mean PMA was 35.3 ± 12.8 cm^2^. Whereas smoking controls had a mean PMA of 43.3 ± 16.6 cm^2^ and non-smoking controls had a mean PMA of 36.4 ± 11.9 cm^2^ (Fig. 1). Likewise, regression modeling of PMA demonstrated participants with very severe COPD were more likely to have lower PMA when compared to both non-smoking controls (β = − 5.9, SE = 1.6, p < 0.001) and smoking controls (β = − 12.8, SE = 0.66, p < 0.001). Sex-stratified regression models exhibited a similar trend. In males, having very severe COPD was significantly associated with lower PMA when compared to both non-smoking controls (β = − 13.7, SE = 2.5, p < 0.001) and smoking controls (β = − 17.7, SE = 0.79, p < 0.001). Likewise, in females, having very severe COPD was significantly associated with lower BMD when compared to both non-smoking controls (β = − 7.8, SE = 1.1, p < 0.001) and smoking controls (β = − 9.0, SE = 0.56, p < 0.001).

### Relationship between PMA and BMD from chest CT in mild to very Severe COPD

PMA was independently associated with BMD (β = 1.40, p < 0.001, r = 0.41) in subjects with mild to very severe COPD, after adjustment for gender, pack years, FEV_1_ percent predicted, BMI category, severe exacerbations, and number of comorbidities. Meaning, a one unit increase in PMA, after adjusting for covariates, was associated with a 1.40 unit increase in BMD. However, PMA and BMD each provide independent information based on their low correlation (r = 0.41, p < 0.001). Likewise, PMA was independently associated with BMD in smoking controls (β = 1.41, p < 0.001, r = 0.40) and non-smoking controls (β = 2.16, p < 0.001, r = 0.39).

### Association of BMD and PMA with functional and clinical outcomes in mild to very severe COPD

We assessed the relationship between CT-derived BMD and, separately, PMA with measures of disease severity (FEV_1_ percent predicted and percent emphysema) and function (handgrip strength and 6MWD) in participants with COPD. After adjustment, each 1 unit increase in BMD (β = − 0.07, 95% CI: (− 0.08, − 0.06), p < 0.001) and PMA (β = − 0.40, 95% CI: (− 0.42, − 0.35) p < 0.001) were associated with reduced percent emphysema (Table 2). Likewise, each 1 unit increase in BMD (β = 0.09, 95% CI: (0.07, 0.10), p < 0.001) and PMA (β = 0.51, 95% CI: (0.44, 0.57), p < 0.001) were associated with higher FEV1 percent predicted and with a greater 6MWD (BMD, β = 0.17, 95% CI: (0. 09, 0.25), p < 0.001; PMA, β = 1.20, 95% CI: (0.83, 1.60), p < 0.001) (Table 2). Higher BMD (β = 0.03, 95% CI: (0.001, 0.05), p = 0.045) and PMA (β = 0.20, 95% CI: (0.10, 0.31), p < 0.001) were associated with increased handgrip strength as well (Table 2). Each 1 unit increase in BMD as associated with increased CRP (β = 0.0038, 95% CI: (0.0012, 0.0065), p = 0.0045) (Table 2).

**Table 2:** BMD and PMA association with clinical outcomes in COPDGene study participants with mild to very severe COPD (N = 4248)

Outcome^1^	BMD			PMA		
	$\beta$	95% CI	p-value	$\beta$	95% CI	p-value
Clinical outcomes						
Handgrip Strength (kg)*	0.03	(0.001, 0.05)	0.045	0.20	(0.10, 0.31)	< 0.001
Emphysema (%)	− 0.07	(− 0.08, − 0.06)	< 0.001	− 0.40	(− 0.42, − 0.35)	< 0.001
6MWD (m)	0.17	(0.09, 0.25)	< 0.001	1.20	(0.83, 1.60)	< 0.001
FEV1pp	0.09	(0.07, 0.10)	< 0.001	0.51	(0.44, 0.57)	< 0.001
CRP (mg/dL)^**^	0.0038	(0.0012, 0.0065)	0.0045	− 0.012	(− 0.02, 0.0007)	0.064

BMD, bone mineral density; BMI, Body Mass Index; CI, confidence interval; COPD, chronic obstructive pulmonary disease; CRP, C-Reactive Protein; FEV1pp, forced expiratory volume in 1 s percent predicted; PMA, pectoralis muscle area; KG, Kilograms; 6MWD, 6-min walk distance (m)

^1^Models were adjusted for pack years, sex, number of comorbidities (cancer, diabetes, heart disease, and gastroesophageal reflux disease), severe exacerbations, and categorical BMI. 6MWD was additionally adjusted for height

*Handgrip strength was only available in a small subset of participants with COPD (N = ???)

**CRP was only available in a small subset of participants with COPD (N = 347)

### Relationship between BMD and PMA derived from chest CT and survival in mild to very severe COPD

**Fig. 2:**
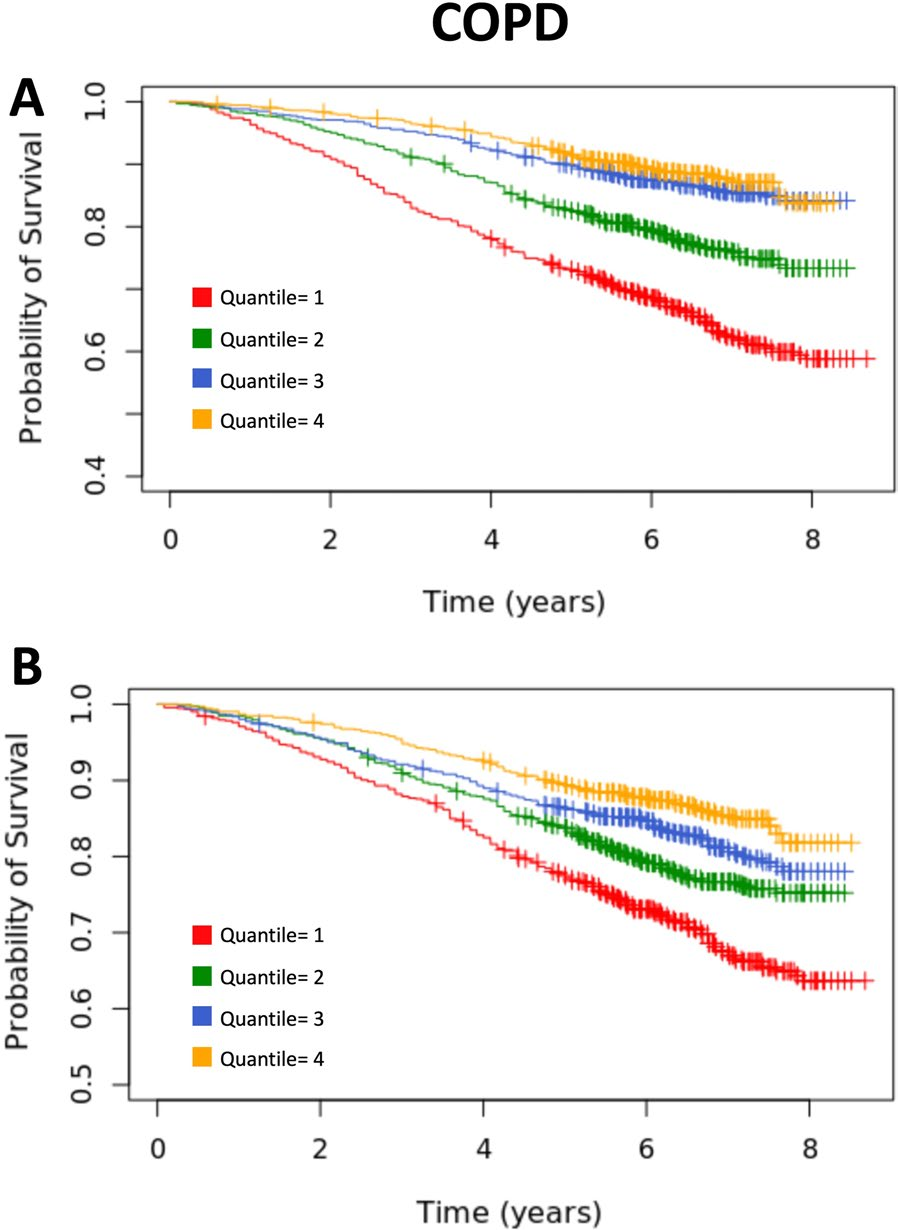
Kaplan–Meier curves for all-cause mortality by sex-specific quartiles of PMA cm^2^ (**A**) and BMD mg/cm^3^ (**B**) in participants with mild to very severe COPD. Sex-specific quartiles of BMD and PMA were calculated using non-smoking controls. The average length of follow-up time was 6.3 years. BMD, Bone Mineral Density; COPD, Chronic Obstructive Pulmonary Disease; PMA, Pectoralis Muscle Area

The duration of follow-up in the present survival analyses was approximately 8.6 years with an average follow-up time of 6.3 years. Over the duration of follow-up, 798 deaths occurred (21.6% of COPD participants with survival data). COPD participants with the highest quartiles of BMD (Table 3: model 1) and PMA (Table 3: model 2) had the best survival in comparison with the lowest quartiles. More specifically, the highest BMD quartile, BMD ≥ 143.6 mg/cm^3^ in men and ≥ 142.1 mg/cm^3^ in women, was associated with a 36.0% (HR = 0.64, 95% CI: 0.52–0.80, p < 0.001) decreased risk of death compared to the lowest quartile, BMD < 87.3 mg/cm^3^ in men and BMD < 87.1 mg/cm^3^ in women (Table 3, model 1). Similarly, the highest PMA quartile, PMA ≥ 47.9 cm^3^ in men and PMA ≥ 30.5 cm^3^ in women, was associated with a 46% (HR = 0.54, 95% CI = 0.43–0.68, p < 0.001) decreased risk of death compared to the lowest PMA quartile, PMA < 33.5 cm^3^ in men and PMA < 21.4 cm^3^ in women (Table 3, model 2). When BMD and PMA were included in the same survival model (Table 3: Model 3), the highest quartiles of BMD and PMA were associated with a 25% (HR = 0.75, 95% CI = 0.60–0.93, p = 0.0089) and 42% (HR = 0.58, 95% CI = 0.46–0.73, p < 0.001, Fig. 2) decreased risk of death compared to the lowest BMD and PMA quartiles, adjusting for covariates (Table 3, model 3, Fig. 2). Additionally, the inclusion of BMD and PMA in the same survival model improved overall model fit (r = 0.74, Table 3), accounting for increased variance than BMD and PMA alone (r_BMD_ = 0.72, r_PMA_ = 0.73, Table 3).

**Table 3:** Relationship between risk of all-cause mortality with BMD or PMA Quartiles in COPDGene study participants with mild to very severe COPD (N = 4248)

Model^1^	Hazard Ratio	95% CI	p-value	r
Model 1: BMD				0.72
Highest vs Lowest	0.64	0.52–0.80	< 0.001	
3rd quartile vs Lowest	0.77	0.63–0.93	0.0072	
2nd quartile vs Lowest	0.81	0.68–0.96	0.026	
Model 2: PMA				0.73
Highest vs Lowest	0.54	0.43–0.68	< 0.001	
3rd quartile vs Lowest	0.52	0.42–0.64	< 0.001	
2nd quartile vs Lowest	0.78	0.66–0.93	0.0058	
Model 3: BMD and PMA				0.74
BMD				
Highest vs Lowest	0.75	0.60–0.93	0.0089	
3rd quartile vs Lowest	0.83	0.68–1.01	0.062	
2nd quartile vs Lowest	0.85	0.71–1.02	0.089	
PMA				
Highest vs Lowest	0.58	0.46–0.73	< 0.001	
3rd quartile vs Lowest	0.54	0.44–0.67	< 0.001	
2nd quartile vs Lowest	0.79	0.67–0.95	0.012	

BMD, bone mineral density; BMI, body mass index; CI, confidence interval; COPD, chronic obstructive pulmonary disease; FEV1, forced expiratory volume in 1 s; PMA, pectoralis muscle area

^1^Adjusted for pack years, gender, FEV1 percent predicted, categorical BMI, categorical comorbidities, and severe exacerbations

## Discussion

We demonstrated that increased BMD and PMA measured by chest CT are associated with reduced morbidity and mortality in participants with mild to very severe COPD from a large cohort. The relationships between increased BMD and PMA measured using chest CT with improved clinical and functional outcomes mirror what has been reported for BMD and FFM measured by DEXA [24, 29, 37]. Further, our findings indirectly support the role of bone muscle crosstalk in COPD as survival models containing both BMD and PMA revealed intermediate phenotypes with intermediate hazards.

On average, higher BMD and PMA were correlated with reduced COPD severity. Participants with normal spirometry had a higher mean BMD and PMA compared to non-smoking controls. One explanation for this finding could be the difference in sex distribution between non-smoking controls (30.8% male) and participants with COPD (56.3% male) and normal spirometry (53.3% male). In addition, the relationship between increased lung function and higher BMD and PMA could be explained, in part, by increased inflammation and decreased physical activity in COPD [38]. Systemic inflammation, a hallmark of COPD, is a risk factor for osteoporosis that has been shown to increase with worsening disease severity [38]. Further, COPD patients have been shown to be more physically inactive compared to age-matched controls with normal lung function [38].

In the current study, COPD subjects with higher BMD tended to have higher CRP. It is important to note CRP was only available in a small number of subjects with COPD (N = 347). Although the result was statistically significant, each 1 unit increase in BMD increased only a small magnitude of the CRP (0.00038 mg/dL). Normal to minor elevation in CRP is defined as 0.3 to 1.0 mg/dL [39]. Nonetheless, the finding is surprising because inflammation upregulates osteoclasts and downregulates osteoblasts, which should increase bone resorption [40]. It is likely underlying co-morbidities prevalent in COPD may be driving the finding. For example, being overweight or obese has been associated with increased mechanical loading and higher bone density [41]. In the current study, COPD subjects were more likely to be overweight or obese thus this is one possible explanation for higher BMD being associated with higher CRP.

In COPD, skeletal muscle dysfunction affects both respiratory and limb muscles with limb muscles largely the focus of sarcopenia treatment and management in COPD [42]. Limb muscle dysfunction is defined as a reduction in either muscle strength or function (or both), and sarcopenia is defined as a reduction in both muscle strength and function [43]. To our knowledge, there has not been a direct investigation into the relationship between PMA and limb muscle dysfunction, however, we have demonstrated PMA can be used to accurately derive FFMI [24, 28]. In COPD, FFMI is highly correlated with muscle strength, but not muscle function [44]. Lower muscle mass does not always lead to impairment of function [45], therefore, we would not expect to see a perfect correlation between PMA and limb muscle dysfunction as this relationship is non-linear [46]. However, monitoring muscle strength is important as reduced muscle strength is independently associated decreased exercise and functional capacity in COPD [47]. Monitoring skeletal muscle could lead to earlier interventions to prevent disability in COPD.

As COPD is irreversible and treatments such as strength training, nutritional management, and supplementation do exist for osteoporosis and sarcopenia, early screening for and treatment of these comorbidities by pulmonologists may improve risk stratification and quality of life for patients with COPD [7, 9, 15–19]. A recent study demonstrated an association between longitudinal loss of PMA and increased mortality, independent of BMI or initial muscle mass [48]. We observed the same trend in survival analyses of PMA alone and expanded upon these findings by demonstrating that incorporating both BMD and PMA in survival models improves overall model fit. Additionally, our previous research has demonstrated PMA can be used to estimate FFMI, as well as monitor changes in PMA over time [24, 48]. Currently, neither PMA nor BMD assessed on chest CT are replacements for their quantification using DEXA. The purpose of our study was to demonstrate the utility of available BMD and PMA measurements as harbingers of sarcopenia and osteoporosis which are underdiagnosed in pulmonary populations. Ideally, adverse changes in BMD and PMA on chest CTs could be assessed as part of standard of care serving as an indicator for referral for further screening via DEXA. Increasing awareness of tools for monitoring extrapulmonary conditions complement current practices in the pulmonary setting to improve outcomes for COPD patients. Currently, DEXA is the gold standard for diagnosis and prescribed treatment plans for osteoporosis [7, 49]. Although it can be used to assess muscle mass, it is not routinely available for clinical practice. Additional work to demonstrate its value in assessing sarcopenia are needed to integrate it into practice.

A strength of our research is COPDGene is a large study with deep phenotyping of COPD-related characteristics, representative of clinical centers across the United States. Another strength of our study was that PMA and BMD were manually derived from chest CT in close to 10,000 participants, which was a significant undertaking necessary because of lack of automation. A limitation of our study was the size of the never smoker group used to make comparisons to the subjects in the COPD and normal spirometry smoking groups. Despite this limitation, our findings were robust to sex-stratified analyses aimed at mitigating potential biases due to differences in sex distribution. Further, handgrip strength is known to vary not only by COPD status, but also based on sex, age, and smoking history [50]. In the current study, handgrip strength was only available in a small subset of the total cohort (N = 418) and was not significantly different between COPD cases and smoking controls. As women typically have lower HGS than men, we further explored this relationship in stratified analyses. In sex stratified analyses, smoking controls had a higher mean HGS than subjects with COPD (Additional file 1: Fig. S1). Another limitation of our study was that although BMD and PMA are associated with measures of disease severity and mortality in COPD, we have not demonstrated the measures can be used to diagnose osteoporosis and sarcopenia. This will require additional research.

Overall, low BMD and PMA are associated with osteoporosis and sarcopenia. These comorbidities substantially increase economic burden and significantly reduce quality of life and survival in COPD. Our findings highlight the importance of monitoring these extrapulmonary conditions and providing opportunities for early screening in the pulmonary setting to improve the standard of care for COPD patients. However, additional research is still necessary to evaluate the sensitivity of using BMD and PMA as a surrogate to diagnose osteoporosis and sarcopenia.

## Supplementary Information

**Additional file 1: Figure S1.** Sex-stratified Clinical and Functional Measures in (A) Females and (B) Males with handgrip strengthdata. Values in red correspond to mean handgrip strength. * Corresponds to a statistically significant (p< 0.05)difference between participants with COPD and smoking controls.

## Data Availability

The datasets used and analyzed during the current study are available from the corresponding author on reasonable request.
