# Supplementary material for: Increased chest CT derived bone and muscle measures capture markers of improved morbidity and mortality in COPD
Source: Respir Res. 2022 Nov 15;23:311. doi: 10.1186/s12931-022-02237-w (PMC9664607; doi:10.1186/s12931-022-02237-w)
Supplement: Supplementary file 1 — Additional file 1: Figure S1. Sex-stratified Clinical and Functional Measures in (A) Females and (B) Males with handgrip strengthdata. Values in red correspond to mean handgrip strength. * Corresponds to a statistically significant (p< 0.05)difference between participants with COPD and smoking controls. [file 12931_2022_2237_MOESM1_ESM.pdf]

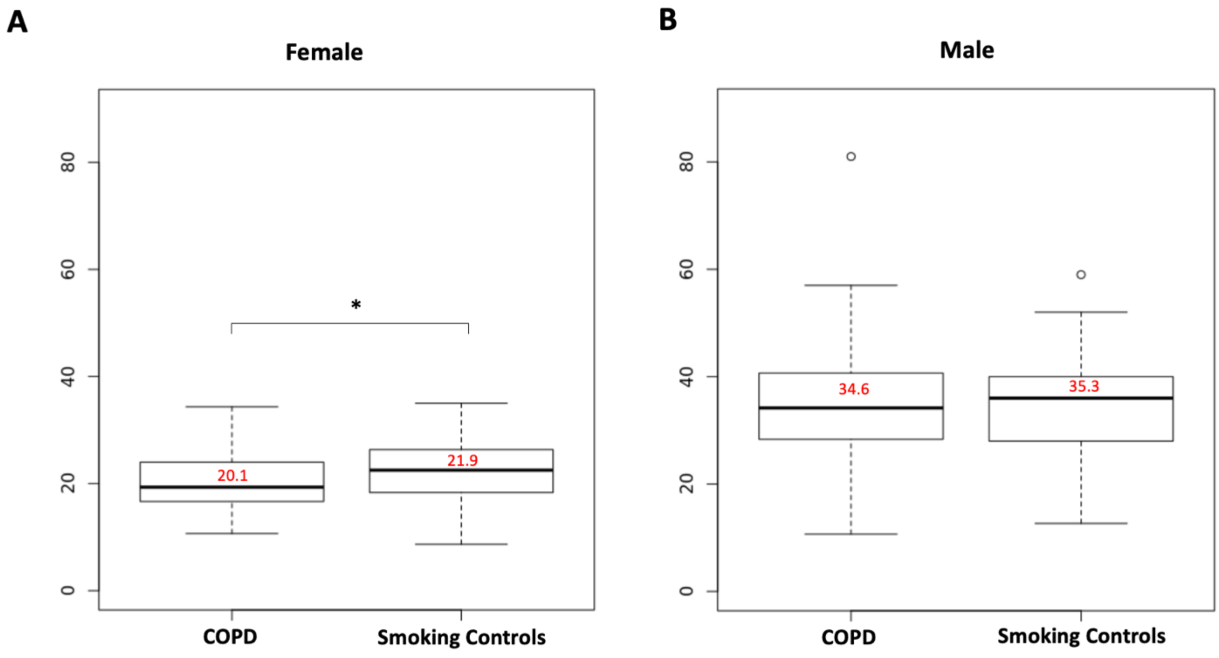

Figure S1. Sex-stratified Clinical and Functional Measures in **(A)** Females and **(B)** Males with handgrip strength data. Values in red correspond to mean handgrip strength. * Corresponds to a statistically significant (p< 0.05) difference between participants with COPD and smoking controls.
